# Mitochondrial Dysfunction of Astrocytes Mediates Lipid Accumulation in Temporal Lobe Epilepsy

**DOI:** 10.14336/AD.2023.0624

**Published:** 2024-05-07

**Authors:** Yang Su, Meng Tang, Minjin Wang

**Affiliations:** ^1^Department of Laboratory Medicine, West China Hospital of Sichuan University, China.; ^2^Department of Neurology, West China Hospital of Sichuan University, China.

**Keywords:** temporal lobe epilepsy, astrocytes, mitochondrial dysfunction, lipid accumulation, neuroinflammation

## Abstract

Lipid-accumulated reactive astrocytes (LARAs) have recently been confirmed to be a pivotal cell type present in temporal lobe epilepsy (TLE) lesions. These cells not only induce anomalous lipid accumulation within the epileptic foci but also decrease the seizure threshold by employing upregulated activation of the adenosine A2A receptor (A_2A_R). Furthermore, disturbances in mitochondrial oxidative phosphorylation (OxPhos) have been noted as significant drivers of lipid accumulation in astrocytes. Moreover, the deficiency of OxPhos in astrocytes can induce severe neuroinflammation, which can worsen the progression of TLE. Accordingly, further exploration of the correlation between mitochondrial dysfunction, LARAs-mediated lipid accumulation, and A_2A_R activation within epilepsy lesions is warranted. It could potentially elucidate the vital role of mitochondrial dysfunction in the pathogenesis of TLE.

Epilepsy stands as a prominent neurological disorder, encompassing a considerable prevalence among individuals. Among them, temporal lobe epilepsy (TLE) occupies a considerable proportion. Remarkably, nearly 30% of patients diagnosed with this condition inevitably encounter a formidable challenge known as drug-resistant epilepsy [1, 2]. The two primary histopathological characteristics associated with TLE manifest as neuronal demise and the development of glial scars within the epileptogenic focus. Reactive astrocytes constitute the principal components of glial scars [3, 4]. The specific role of reactive astrocytes in TLE, indeed, remains a fascinating question, as our understanding of their functions in this context remains limited.

On March 20, 2023, a research paper titled "*Lipid-accumulated reactive astrocytes promote disease progression in epilepsy*" was published online, led by Yan Chao and his team. This groundbreaking study shed light on the molecular characteristics and biological functions of a newly identified subtype of reactive astrocytes called "lipid-accumulated reactive astrocytes" (LARAs), both in patients and mouse models with TLE. The study made a significant discovery regarding the presence of abnormal lipid accumulation in epileptic foci, with astrocytes showing more pronounced lipid accumulation compared to neurons. Additionally, the researchers found that lipid metabolism-related signaling pathways were notably activated in astrocytes. By employing single-cell RNA sequencing, they identified the high expression of the APOE gene in LARAs, which plays a crucial role in mediating lipid transport from neurons to astrocytes in an APOE-dependent manner. Further mechanistic investigations revealed that LARAs facilitated the progression of TLE by upregulating the expression of adenosine A2A receptors (A_2A_R) on astrocytes. This upregulation, in turn, hindered glutamate reuptake and promoted neuronal discharges. This finding provided valuable insights into the underlying mechanisms by which LARAs exacerbate the development of TLE. The research paper by Yan Chao et al. represents a significant milestone in understanding the intricate involvement of reactive astrocytes, particularly LARAs, in the pathogenesis of TLE [5, 6].

The research conducted in this study provided a comprehensive characterization of the molecular phenotypes and functional properties of reactive astrocytes within the focal areas of patients with TLE. Moreover, it elucidated the significant influence of lipid accumulation in astrocytes on TLE, showcasing the upregulation of the APOE gene and A_2A_R expression as key factors in this process. These findings made a notable contribution to the understanding of the involvement of abnormal lipid metabolism in the development of TLE and its underlying pathogenesis [6]. However, we believe that there are some room worth further consideration and exploration in this research. While abnormal lipid accumulation has been confirmed in the brain tissue of both TLE patients and epileptic mice, the precise reason behind this abnormal lipid accumulation in astrocytes within epileptic foci remains unknown. Understanding the underlying mechanisms contributing to this phenomenon would be valuable for a comprehensive understanding of TLE pathology. Additionally, the significant upregulation of A_2A_R in LARAs, leading to increased neuronal excitability and the progression of epilepsy, raises intriguing questions regarding the specific inducers responsible for this upregulation. Identifying these inducers would provide important cognition of the regulatory mechanisms involved in A_2A_R expression within reactive astrocytes. Further investigation and exploration are necessary to address these questions and enhance our understanding of the complex dynamics at play in TLE.

Fatty acids (FAs) are crucial components of lipids and the main focus of lipid metabolism in both neurons and astrocytes [7]. The accumulation of excessive free fatty acids (FFA) has potent toxic effects on cells. To mitigate this risk, FAs are typically stored in the form of triglycerides within lipid droplets. The formation of lipid droplets serves as an effective protective mechanism, safeguarding against the harmful consequences of lipid toxicity induced by FFA [8, 9]. The mitochondria play a vital role in the degradation of FAs. When FAs are stored within lipid droplets, they are primarily metabolized through a process called mitochondrial β-oxidation. β-oxidation is a metabolic process responsible for the breakdown of FAs into shorter FAs chains and acetyl-CoA while generating energy. In the cytoplasm, FAs are first converted to acyl-CoA and then esterified with choline to form fatty acetylcholine, which is subsequently transported into the mitochondria. Within the mitochondria, fatty acylcholine undergoes stepwise β-oxidation, producing acetyl-CoA and shorter FAs chains, and generating a large amount of energy [10]. The efficient functioning of mitochondrial β-oxidation is essential for the proper metabolism and utilization of FAs as an energy source in cells [11-13]. Moreover, the complete degradation of FAs necessitates the involvement of the mitochondrial tricarboxylic acid (TCA) cycle and oxidative phosphorylation (OxPhos). The TCA cycle plays a pivotal role in the oxidation of acidic carbon atoms present in nutrients such as glucose, FAs, and amino acids into carbon dioxide and water. In the process, electron donors such as NADH and FADH2 are generated. During OxPhos in the mitochondria, these electron donors release electrons and protons through a series of enzymes in the electron transport chain, thereby creating a proton gradient. This gradient drives the rotation of the adenosine triphosphate (ATP) synthase rotor, leading to the generation of ATP by combining ADP and inorganic phosphate [14, 15]. These two essential processes operate within the mitochondria to facilitate the breakdown of FAs-derived acetyl-CoA. The acetyl-CoA generated from β-oxidation enters the TCA cycle, where it undergoes further enzymatic reactions, leading to the production of NADH and FADH2. These reduced coenzymes serve as crucial electron carriers that participate in OxPhos, which locates in the mitochondrial inner membrane. Through OxPhos, the electron transport chain utilizes the energy derived from NADH and FADH2 to generate ATP, the primary energy currency of cells. Therefore, the collaborative functioning of mitochondrial β-oxidation, the TCA cycle, and OxPhos are indispensable for the complete degradation of FAs and the efficient production of cellular energy [16].

Under normal conditions, there exists a crucial lipid metabolic coupling between neurons and astrocytes, which has a great impact on maintaining the homeostasis of lipids in the brain [7]. High-intensity neuronal activity can readily result in the accumulation of FAs; however, neurons lack the inherent capacity to independently metabolize FAs. This limitation primarily stems from the insufficient antioxidant defense mechanisms within neurons, making it challenging for them to withstand the stimulation of reactive oxygen species (ROS) generated during the process of FAs β-oxidation [17]. Furthermore, neurons have a limited ability to synthesize lipid droplets for the storage of FAs. Consequently, the accumulation of ROS can trigger peroxidation reactions of free FAs within neurons [7]. This disruption in turn can compromise the integrity of cell membranes and mitochondrial membranes, thereby exacerbating the harmful effects of lipid toxicity induced by FAs on neurons [18]. Despite their lower abundance in astrocytes, mitochondria are critical targets for FAs metabolism in the central nervous system (CNS). This is primarily attributed to the buffering and breakdown capacity of astrocytes for FAs, as well as their superior antioxidant capacity compared to neurons. Unlike neurons, astrocytes can produce copious lipid droplets for storing FAs, which provides a crucial foundation for undertaking extensive FAs degradation tasks. Any unutilized and unprocessed FAs are stored in lipid droplets, which can effectively buffer the toxicity of accumulated FFA in astrocytes that have not yet been degraded within a short period. Importantly, astrocytes are equipped with numerous antioxidative factors that can effectively combat oxidative stress resulting from FAs β-oxidation. This ability can effectively alleviate the detrimental effects of lipid toxicity in astrocytes [7, 19]. Consequently, the proper functioning of mitochondria in astrocytes assumes significant responsibility for the degradation of FAs within the brain. The maintenance of lipid homeostasis in both neurons and their surrounding environment is heavily reliant on the pivotal role played by astrocytes [17]. Studies have demonstrated that the accumulation of FAs and the generation of oxidative stress signals in activated neurons can trigger the formation of lipid droplets in neighboring astrocytes [20].

Additionally, highly activated neurons can transfer FAs to lipid droplets in astrocytes through APOE-positive lipid particles. This process helps mitigate the toxic effects of FAs accumulation by facilitating their metabolism through mitochondrial β-oxidation and promoting the expression of detoxification genes within astrocytes [7]. Indeed, the significant impact of APOE expression in LARAs on the process of lipid transfer from neurons to astrocytes has also been demonstrated by Yan Chao and colleagues. The high expression of APOE in LARAs exerts an important impact on facilitating the efficient transfer of lipids from neurons to astrocytes [5]. In the brain, APOE protein is primarily synthesized by astrocytes and acts as the principal carrier for lipid transport. Moreover, FAs from neurons are typically transported to astrocytes in an APOE isoform-dependent manner. The distinct subtypes of APOE can impact the efficacy of lipid transport in neurons and astrocytes. Notably, APOE4 shows significantly lower efficiency in stimulating neuronal lipid transport and astrocytic mitochondrial oxidative phosphorylation, when compared to APOE3 [21].

It is worth noting that a recent study published in *Nature Metabolism* on March 24, 2023, sheds new light on the essential role of astrocyte mitochondrial OxPhos in the degradation of FAs and the maintenance of lipid homeostasis in the brain. The study provided direct evidence that abnormal mitochondrial OxPhos function can result in lipid accumulation in astrocytes, which can in turn impair the normal lipid metabolic coupling between neurons and astrocytes. Ultimately, this disruption can have a detrimental effect on neuronal lipid homeostasis [22]. Indeed, while the research primarily focused on neurodegenerative diseases like Alzheimer's disease, it effectively demonstrated the essential nature of astrocytes' mitochondrial OxPhos in FAs degradation. This underscores the significance of normal mitochondrial respiration function in maintaining lipid homeostasis within the brain. Moreover, the study validated that OxPhos deficiency in astrocytes can stimulate the expression of Interleukin-3 (IL-3), inducing microglial activation and promoting the release of inflammatory factors like Interleukin-1β (IL-1β) and Tumor Necrosis Factor (TNF), which triggers severe neuroinflammatory responses.

There is ample evidence highlighting the significance of neuroinflammation in the reduction of seizure threshold and the progression of epilepsy [3, 23-26]. This provides further support for the hypothesis that mitochondrial OxPhos dysfunction is a crucial underlying factor in the pathogenesis and progression of TLE. Activated astrocytes and microglia are the primary contributors to neuroinflammation. The promotion of inflammation in TLE is mainly attributed to the release of inflammatory cytokines and activation of related inflammatory pathways by glial cells. After the onset of an inflammatory response, aberrantly activated glial cells respond further to inflammatory signals and modify their normal regulatory effects on neuronal network excitability, thereby promoting seizure onset and recurrence in TLE [3, 27].

Following epileptogenic injury events, stimulated astrocytes undergo significant transcriptional changes mediated by transforming tumor growth factor-β (TGF-β), which exacerbates downstream smad2/3 phosphorylation. TGF-β activation induces the production of seizure-related cytokines, such as IL-1β [28, 29]. TGF-β signaling also increases the mRNA levels of glial fibrillary acidic protein and neurocan in astrocytes, confirming the crucial role of TGF-β in inflammation-related changes during epileptogenesis [29, 30]. The activation of the TGF-β pathway has a significant impact on epileptic seizures. In vitro, experiments have demonstrated that TGF-β can stimulate the formation of glial scars in astrocytes and mediate neuronal network hyperexcitability. TGF-β signaling is involved in the generation of reactive excitatory synapses and the breakdown of inhibitory neuron networks, favoring the pathological hyperexcitability of neuronal networks [31]. Studies have shown that blocking the activation of astrocytic TGF-β receptors can effectively control seizures in the pilocarpine-induced rat model of epilepsy. Moreover, specific TGF-β inhibitors significantly reduce the frequency of spontaneous seizures in experimental animals [32].

The activation of the high mobility group box 1 (HMGB1) cascade immune pathway is another crucial evidence for the involvement of astrocytes in epileptic neuroinflammation [3, 26]. Inflammatory cytokines such as IL-1β, TNF-α, and oxidative stress products like ROS are significant activators of HMGB1. These stimuli induce the cytoplasmic translocation of HMGB1, which is passively released into the extracellular space, making it a crucial mediator of epileptic neuroinflammation [33]. HMGB1 released from activated neurons or astrocytes upregulates the expression of Toll-like receptor 4 (TLR4) and receptor for a receptor for advanced glycation end-products (RAGE). The activation of the HMGB1-TLR4 inflammatory signaling pathway effectively mediates signal transduction downstream of epileptic foci [34]. These abnormal immune activations alter the storage and release of glutamate by astrocytes, suppress the release of γ-aminobutyric acid, upregulate neuronal network excitability, and increase the likelihood of abnormal discharge [26, 35]. To some extent, the use of antioxidants can control the status epilepticus in rats by blocking the production of ROS and preventing the cytoplasmic translocation of HMGB1 in hippocampal neurons and glial cells [36]. Pre-injection of anti-HMGB1 monoclonal antibodies can effectively inhibit the cytoplasmic translocation of HMGB1 and significantly reduce the frequency of seizure acid in epileptic mice induced by kainic [37].

In addition to astrocytes, the aberrant activation of microglia is a crucial factor in the perpetuation of neuroinflammation in TLE [38, 39]. Research conducted by Marschallinger et al. has confirmed that under specific conditions, microglia also have significant lipid droplet accumulation, which is closely associated with neuro-inflammatory reactions. The potent pro-inflammatory mediator, lipopolysaccharide (LPS), acts as a ligand for TLR4 and induces the formation of lipid droplets in microglia. Consequently, microglia with lipid droplet accumulation produces high levels of ROS and excessively release various cytokines, such as TNF-α, IL-1β, and interleukin-6 (IL-6). The vicious cycle between lipid accumulation and inflammatory reactions leads to microglia being in a state of overactivation [40]. Abnormally activated microglia and astrocytes also directly release substantial amounts of pro-inflammatory molecules, such as IL-6 and prostaglandins, which promote gliosis and epileptic seizures by altering calcium ion flow in neural networks, glutamate uptake, and inhibit long-term potentiation (LTP) and hippocampal neurogenesis [25].

In our view, the role of mitochondrial dysfunction in the pathogenesis of TLE does deserve deep consideration. It is plausible to hypothesize that mitochondrial dysfunction could potentially contribute to abnormal lipid accumulation by impeding FAs degradation in astrocytes located in epileptic foci. Furthermore, mitochondrial dysfunction may enhance the expression of inflammatory signals, inducing severe neuroinflammation, leading to the exacerbation and persistence of epileptic seizures.

Indeed, there is a correlation between abnormal mitochondrial function and the expression of A_2A_R that has been observed in various disease processes, especially in cardiac ischemia/ reperfusion injury. In conditions where mitochondrial energy metabolism is impaired, a substantial accumulation of ROS occurs. Excessive ROS levels can induce membrane lipid peroxidation, leading to direct damage to the mitochondrial structure. Moreover, the release of pro-apoptotic signals, such as cytochrome C, can activate a cascade of cell death reactions. In this context, the activation of A_2A_R has been shown to have a protective effect. By blocking ROS production and promoting ROS degradation through the increased activity of peroxidases, A_2A_R can mitigate oxidative stress injury. This process also inhibits the opening of the mitochondrial permeability transition pore (mPTP), thereby reducing mitochondrial swelling and minimizing oxidative stress-induced damage [41]. Furthermore, it has been demonstrated that the A_2A_R agonist NECA can effectively reduce the loss of mitochondrial membrane potential and inhibit excessive mitochondrial matrix calcium (Ca^2+^) accumulation. This mechanism is effective in diminishing oxidative stress-induced mitochondrial damage. NECA's ability to modulate these processes highlights its potential as a protective agent in protecting mitochondrial normal functions under conditions of oxidative stress [42, 43]. These observations suggest that the activation of A_2A_R may be in favor of reducing mitochondrial dysfunction-related oxidative stress injury and preserving mitochondrial function. It is intriguing to consider whether a similar mechanism involving A_2A_R-mediated ROS modulation and mitochondrial protection could potentially contribute to the correlation between abnormal mitochondrial function and lipid metabolism in epileptic brain tissues. Further research is warranted to explore this possibility and shed light on the interplay between A_2A_R, mitochondrial function, and lipid metabolism in the context of TLE.

A_2A_R is also present in the CNS. Mitochondria are the main energy source and energy metabolism regulation points for normal CNS functions. Therefore, it is logical to assume that the upregulation and activation of A_2A_R in neurons and surrounding cells may be a compensatory protective mechanism against oxidative stress caused by mitochondrial damage. In the case of epileptic brain tissue, studies have found that A_2A_R expression is upregulated in LARAs. However, A_2A_R expression in astrocytes can increase neuronal activity and affect glutamate reuptake. As a result, this compensatory mechanism, which may be secondary to mitochondrial damage, can further increase neuronal excitability and susceptibility to epileptic seizures [6, 44]. Further speculation indicates that when the compensatory protective effects of A_2A_R are ineffective, the disruption of ion homeostasis resulting from mitochondrial dysfunction and the influence of A_2A_R on abnormal neuronal discharges may further reduce the seizure threshold. In such circumstances, the combined effects of impaired mitochondrial function and dysregulated A_2A_R activity can exacerbate the propensity for seizures to occur.

The proper functioning of mitochondria is vital in the regulation of epileptic seizures. The ATP production process through mitochondrial OxPhos has a substantial impact on maintaining ion homeostasis, which eventually affects the generation of normal action potentials and synaptic transmission in neurons. Seizures, however, impose significant energy demands on the CNS. Consequently, any factors that contribute to mitochondrial dysfunction can potentially lower the threshold for seizure activity [45, 46]. Gábor Zsurka and colleagues have clarified the role of secondary mitochondrial dysfunction in the development of epilepsy. Their review suggests that mitochondrial dysfunction, which leads to an energy crisis specifically within neurons, can promote the progression of epilepsy. This dysfunction is characterized by the failure of OxPhos and the subsequent accumulation of ROS. These pathological processes can disrupt Ca^2+^ homeostasis, impede ion pump activity, and impair the normal function of ion channels. Ultimately, these disturbances can contribute to the occurrence of epileptic seizures [47].

## Summary

The study by Yan Chao et al. presents significant findings regarding the role of lipid accumulation in astrocytes in TLE. They highlight the high expression of A_2A_R in LARAs, which influences neuronal excitability and increases susceptibility to discharge. The article also emphasizes the need for further exploration into the factors that induce lipid accumulation and the regulatory role of LARAs on A_2A_R expression. In parallel, the concurrent article by Yashi Mi and colleagues elucidates the impact of mitochondrial OxPhos dysfunction on astrocytes responsiveness and normal functions. They demonstrate that mitochondrial dysfunction is a considerable contributing factor to lipid accumulation and neuroinflammation. Moreover, evidence suggests that the upregulation of A_2A_R expression may serve as a compensatory protective mechanism in the face of mitochondrial energy metabolism dysfunction. These research findings inspire investigating the impact of abnormal lipid metabolism on TLE by examining mitochondrial structure and functions. The aim is to gain a comprehensive understanding of the underlying mechanisms involved in the pathogenesis of TLE. Further exploration in this area has the potential to unravel the complexities surrounding abnormal lipid metabolism and its implications for TLE.
